# The COL5A1 Gene Allelic Combination and ACL Injury Risk in Team Sport: A Preliminary Report

**DOI:** 10.1055/s-0043-1771531

**Published:** 2024-04-22

**Authors:** Carla Maria Calò, Myosotis Massidda, Roberto Sorge, Alessandra Tiloca, Giovanni Monteleone

**Affiliations:** 1 Departamento de Ciências Biológicas e Ambientais, Universidade de Cagliari, Itália; 2 Faculdade de Medicina e Cirurgia, Universidade de Cagliari, Itália; 3 Departmento de Sistemas Médicos, Faculdade de Medicina e Cirurgia, Universidade de Roma “Tor Vergata”, Roma, Itália; 4 Universidade de Roma “Tor Vergata”, Roma, Itália; 5 Escola de Educação Física e Ciências do Exercício, Universidade de Roma “Tor Vergata”, Roma, Itália

**Keywords:** anterior cruciate ligament, athletes, athletic injuries, polymorphism, single nucleotide, rupture

## Abstract

**Objective:** The aim of this study was to examine the relationship between BstUI restriction fragment length polymorphisms (RFLP) C/T (rs 12722) and DpnII RFLP B1/B2 (rs 13946) COL5A1 polymorphisms and the anterior cruciate ligament (ACL) rupture in competitive team-sport athletes.

**Methods** Sixty-eight team-sport players (n = 36 women and n = 32 men) with non-contact ACL rupture (ACLR) occurred during sport practices (ACLR Group) and 42 healthy players (n = 20 women and n = 22 men) (Control Group) participated in the study. Genomic DNA was extracted from buccal swab with salting out method. All samples were genotyped for the polymorphisms rs12722 and rs13946 by polymerase chain reaction (PCR) and restriction enzymes analysis.

**Results** No significant difference has been found between ACRL and Control groups in age, height, weight body, mass index, sport practice (hours/week) and gender distribution among the different team sports. Control group had longer sport careers ( *p* < 0.005). The frequency distributions of COL5A1 DpnII nucleotide polymorphisms were in Hardy-Weinberg equilibrium (HWE) in both groups ( *p* of the Hardy-Weinberg (HW) -test > 0.005). Genotype frequencies of COL5A1 BstUI RFLP C/C was lower in the ACLR group compared to the Control group ( *p* of the HW-test = 0.001). Combined CC, B1B1 genotypes showed a protective effect against ACL rupture (OR = 83.3 / 16.7 = 5).

**Conclusions** The COL5A1 gene may be one of the genetic factors associated with ACLR in team sport.

## Introduction

Anterior cruciate ligament rupture (ACLR) is a frequent disabling injury among athletes, that causes knee joint instability. In the USA, estimated incidence of ACLR ranges between 100,000 and 200,000 / year. 1 Reconstructive surgery allows sports practice and a better quality of life. In Italy, the incidence of ACL reconstruction surgery is 21-33 rate per year in 100,000 people, with an incidence between 0.16 and 2.04 procedures per 100,000 individuals younger than 15 years. 2 3

Despite surgical ligament reconstruction, about 79% of these patients develop knee osteoarthritis and 20% suffer a new injury within the next 2 years. 4

Sport ACLRs can be contact injuries (with a teammate or opponent), more frequent in contact sports such as soccer or basketball, 5 or non-contact injuries. In a 10-year period of observation, Majewski et al. 6 noted that 60% of sport knee injuries treated in their hospital were volleyball-related ACL injuries. Agel et al. 7 found that 14% of injuries in volleyball occur via non-contact.

Over a 16-year period of collegiate injuries registration, Hootman et al. 8 reported an injury rate per 1000 athlete-exposures of 0.07 and 0.09 for male basket and soccer players. They report 0.23, 0.28 and 0.09 for female basket, soccer and volleyball players respectively.

The risk factors for ACL injury are classified as exogenous (e.g. laying surface) and endogenous like genetic factors. 9 In inheritable collagen disorders, genetics influence laxity of tissues 10 : generalized joint laxity and hyperextension were found to significantly increase the risk for ACL injury in female athletes. 11 Mutations within the COL5A1 gene cause the classic form of Ehlers-Danlos syndrome (EDS) characterized by joint hypermobility involved in sprains, dislocation / subluxation, and early osteoarthritis. 12 As reported by Mokone et al. 13 , the COL5A1 gene contains RFLP of BstUI (rs 12722) and DpnII (rs13946) within its 3'-untranslated region (UTR). The functional COL5A1 specificity protein 1 (Sp1) binding site polymorphism - BstUI RFLP C / T is positively correlated with tendon and ligament injuries, especially in Caucasian subjects. 14 15 Accordingly, a study on soccer players found that the T/T genotype of the COL5A1 BstUI RFLP showed a trend toward a higher severity of musculoskeletal injuries with respect to the individual carriers of the CC genotype. 15

The aim of this study was to correlate the COL5A1 gene BstUI RFLP and DpnII RFLP polymorphisms with ACLR in volleyball, basketball and soccer athletes.

## Materials and Methods

### Subjects

68 Caucasic players (n = 36 women and n = 32 men) with ACLR occurred during sport practices (ACLR Group) and 42 healthy players (n = 20 women and n = 22 men) (Control Group) participated to the study. All of them practiced team-sports (Volleyball, Basketball, and Soccer) in Italian teams. Subjects gave informed consent to participate. To obtain demographic information and data regarding sport practice; each athlete completed a self-administered questionnaire. The ACLR Group (32 athletes practiced Volleyball, 19 Basketball and 17 Soccer) provided information on the mechanisms and date of anterior cruciate ligament injury.

The study was approved by the Institutional Review Board of the University of Cagliari (Prot. PG/2017/1700) and was carried out in accordance with the ethical and humane principles of the research.

### DNA Analysis

Genomic DNA was extracted from buccal swab, performed on the oral mucosa of the cheek, through salting out method, and amplified by standard PCR following the protocol suggested by Galasso et al. 16

PCR products were subjected to two enzymatic digestions: digestion with BstUI produced two fragments for the T allele (351 and 316 bp) and three fragments for C allele (316, 271, and 80 bp); digestion with DpnII produced a unique fragment for B2 (612 bp) and two fragments (412 and 194 bp) for B1. The fragments obtained were separated through an 8% polyacrylamide gels for BstUI and a 2% agarose gel for DpnII and visualized with Syber Safe staining.

### Statistical Analysis

All data were initially entered into an Excel database (Microsoft, Redmond, Washington – United States) and the analysis was performed using the Statistical Package for the Social Sciences Windows, version 15.0 (SPSS, Chicago, Illinois, USA). Descriptive statistics consisted of the mean ± standard deviation (SD) for parameters with gaussian distributions (after confirmation with histograms and the Kolgomorov-Smirnov test).

Comparison among groups was performed with the ANOVA one-way for continuous parametric variables or the Chi-square test or Fisher's exact test (if cells < 5) for frequencies variables.

HWE tests were conducted considering population frequencies (p2 + 2pq + q2 = 1) and then reported in SPSS, Chi-square nonparametric test. Similarly, the G-square test (G ^2^ test) was performed. A *p* value of < 0.05 was considered statistically significant.

## Results

No significant difference has been found between ACRL and Control groups in age, height, weight body mass index, sport practice (hours/week) and gender distribution among the different team-sports). Control Group athletes have longer sport careers ( *p* < 0.005) ( Table 1 ).

**Table 1:** Characteristics of the of the ACL rupture Group (ACLR) and Control Group

	ACLR	Control	*p*
**Number**	**68**	**42**	
**Age (years)**	**27.3 ± 6.4**	**28.5 ± 8.5**	0.406*
**Height (cm)**	**173.0 ± 9.5**	**174.0 ± 11.4**	0.588*
**Weight (kg)**	**70.0 ± 11.8**	**70.2 ± 12.3**	0.945*
** Body mass index (kg/cm ^2^ ) **	**23.30 ± 3.30**	**23.16 ± 2.99**	0.782*
**Gender (n°males/n°females)**	**32 / 36**	**22 / 20**	**0.200^**
**Sports (Volleyball; Basketball; Soccer)**	**32; 19; 17**	**26; 8; 8**	**0.312^**
**SPORT (years)**	**10.8 ± 4.9**	**16.8 ± 8.5**	0.001*
**SPORT (hours/week)**	**7.7 ± 3.2**	**7.8 ± 4.0**	0.799*

Values are expressed as mean ± standard deviation or a number (n) where applicable.

(*) Anova oneway (^) Chi-Square test.

The genotype and allele frequencies of COL5A1 BstUI RFLPC/T and COL5A1 DpnII RFLP B1/B2 are shown in Table 2 . Genotype frequency distributions of COL5A1 DpnII and COL5A1 BstUI RFLP nucleotide polymorphisms meet the HWE in both groups ( *p* . value > 0.05).

**Table 2:** Genotype Frequencies of the ACLR Group and Control Group - Hardy-Weinberg equilibrium verification

BstU	ACLR	Control
**CC**	**58.90**	**22.22**
**CT**	**34.25**	**44.44**
**TT**	**6.85**	**33.33**
**MAF (T)**	**0.2397**	**0.5556**
***p***	**0.3881**	0.3274*
**DpnII**	**ACL**	**Control**
**B1B1**	**71.23**	**75.56**
**B1B2**	**26.03**	**20.00**
**B2B2**	**2.74**	**4.44**
**MAF (B2)**	**0.15**	**0.1444**
***p***	**0.556**	0.2060*

(*) Hardy-Weinberg equilibrium test.

Linkage disequilibrium was tested with LDlink (Machiela and Chanock, 2015) 17 using data from 1000 genomes (1000 Genomes Project Consortium, 2015). The two single nucleotide polymorphisms (SNPs) resulted in a linkage disequilibrium for European populations.

The distribution of the C/C genotype and the C allele of the COL5A1 BstUI RFLP were lower in the ACLR Group compared with the Control Group ( *p* G ^2^ test= 0.001- Table 3 ). No significant differences have been found in the distribution of the DpnII RFLP polymorphism between ACLR Group and controls ( Table 3 ). Finally, the combination of CC + B1B1 genotypes was more frequent in the controls than in the ACLR group, and associated with a protective effect (OR = 83.3 / 16.7 = 5). The TT, B2B2 genotypes were absent among participants.

**Table 3:** Genotype Frequencies of the ACLR Group and Control Group

ACLR		Control	*p*
COL5A1 BstUI% (CC; CT; TT)	23,8;61,0; 79,2 (0.954)	76,2; 39,0; 20,8 (0.368)	0.001°
COL5A1 DpnII% (B1B1; B1B2; B2B2)	60,8;66,7; 50,0 (0.990)	39,2;33, 3; 50,0 (0.497)	0.952°

(°) Chi-Square test.

## Discussion

Although the biological changes underlying the increased risk of ACL sports injury have not yet been find out, the family predisposition of athletes to ACL ruptures is a consolidated knowledge. 18 19

The COL5A1 gene, located at 9q34.2-q34.3, contains 66 exons distributed over 150 kb of gDNA, and it encodes the 1 chain of type V collagen. 20

The COL5A1 gene codes for a protein chain in type V collagen, which is found in ligaments and tendons, and mutations within it are indicated as a cause responsible for the increased risk of ACL rupture. 19 21

We examined the association between COL5A1 rs12722 C / T (BstUI RFLP) and COL5A1 rs13946 B1/B2 (DpnII) polymorphisms individually and as haplotypes with risk of anterior cruciate ligament rupture in male and female athletes competing in contact / noncontact team sports.

Our results show a lower frequency of the COL5A1 BstUI C/C genotype in the ACLR group compared with the Control Group. No significant differences in genotype distribution or allele frequencies of COL5A1 DpnII was observed.

Posthumus et al. 21 found an underrepresented C/C genotype of COL5A1 BstUI RFLP in Caucasian females but not in males with surgically diagnosed ACL ruptures recruited from sports and recreational clubs.

In male recreational skiers, Stępień-Słodkowska et al. 22 noted no significant differences in genotype distribution or allele frequencies of COL5A1 BstUI RFLP C/T (rs 12722) and COL5A1 DpnII RFLP C/T (rs 13946) polymorphisms between the ACLR group and control group. These authors found an underrepresentation tendency of the C-T haplotype in the ACLR group compared to controls.

Luliska-Kuklik et al. 23 found a significant decrease in frequency in the dominant model of the C/C genotype for the COL5A1 rs13946 gene in male professional soccer players with surgically diagnosed primary ACL rupture.

In our study, athletes within the ACLR and control groups were matched for age, height, body weight, BMI, sport, and length of their sport career. The longer is the sport career the higher is the risk of trauma exposure.

In addition to having a higher frequency of the C/C genotype, the athletes in the Control Group have a longer sports career than those of the ACLR Group: with caution due to the small number of subjects, we hypothesize a protective effect of the C/C genotype, especially if associated with B1B1 (CC, B1B1 OR protective = 83.3 / 16.7 = 5).

Likewise, Posthumus et al. 24 found a significant age dependent increase in the distribution of the COL5A1 BstUI RFLP C/C genotype in a group of physical activity male, asymptomatic for musculoskeletal soft-tissue injuries (without a reported history of tendon injuries). According to these authors, prolonging exposure time to extrinsic risk of injury selects individuals who are genetically low risk of injury, who will be found in greater numbers among older asymptomatic subjects than in the younger asymptomatic subjects.

The COL5A1 BstUI RFLP C/C polymorphism may play a role in increasing the length of the sports career of athletes.

It is unclear which phenotypic expression of COL5A1 gene polymorphisms may be associated with an increased risk of ACL rupture.

In EDS, several COL5A1 gene mutations occur concurrently with specific clinical features. 10 The connective tissue of these patients shows structural changes and ligamentous laxity causing different degrees of joint hypermobility.

Hypermobility has been implicated in ACL injury, 25 and lower limb joint proprioception is reduced in those with benign joint hypermobility syndrome 26 : no correlation has been described between the COL5A1 BstUI RFLP and COL5A1 DpnII polymorphisms with an increase in ligamentous laxity. Future investigations should include the search for an association between changes in joint mobility or ligament tension and the presence of these polymorphisms.

In a retrospective genetic case-control association study, O'Connell et al. 27 found the COL5A1 C/C genotype significantly overrepresented in triathlon and ultra-marathon athlete without a history of exercise-associated muscle cramping (EAMC) compared with athletes with a history of EAMC. The efficiency of the knee flexor muscles, the abductors muscles of the hip and trunk stabilizers muscles efficiency prevent ACL injuries. 28

According to Collins and Posthumus, 29 the COL5A1 rs12722 TT genotype is associated with a surplus V collagen production with a higher risk of some musculoskeletal injuries due to collagen fibril structural changes in collagen fibrils and changes in the properties of soft tissues.

Laguette et al. 30 demonstrated greater stability of the COL5A1 mRNA (encoding for α1(V)) in C/T genotype and speculated that small changes in COL5A1 mRNA stability, even if within the normal physiological range, could result in inter-individual variation in fibrillogenesis; therefore, a different vulnerability to musculoskeletal lesions COL5A1-dependent. We theorize that, in the study group (ACLR Group), the frequent exposure of the ACL to abnormal stresses during team sport practice, reveals the relative susceptibility of the ACL of the athletes with C/T genotype.

As reported by Smith et al., 19 tree more genetic factors may be associated with an increase in ligament fragility: 1) TT genotype of the COL1A1 Sp1, binding site polymorphism. The COL1A1 gene encodes a protein chain within type I collagen, a major structural component of ligaments; 2) the AA genotype of the COL12A1 AluI polymorphism (only in women). This gene encodes for protein chains in type XII collagen, which is believed to regulate fibril diameter in ligaments; 3) the chromosomal region 11q22, where several matrix metalloproteinase genes of physiologic mediators of collagen cleavage and removal are located.

Multiple genetic dependency of ACL susceptibility to rupture may suggest scanning changes in multiple genes.

The main limitation of the present work is represented by the small number of athletes included in the study, which has precluded the possibility of analyzing any differences between genders. The development of clinical or laboratory tools which identify subjects at great risk to this common musculoskeletal injury, would ease a greater people awareness of their own exposure to an ACL rupture. Besides it would be useful in settle the best therapeutic choice after primary ACL rupture diagnosis.

## Conclusions

The COL5A1 gene may be one of the genetic factors associated with ACLR in team-sport. COL5A1 C/C polymorphisms provides a protective effect against the ACLR in both sex. Longer sport career linked to an increased frequency of COL5A1 BstUI RFLP C/C.
