# Supplementary material for: The COL5A1 Gene Allelic Combination and ACL Injury Risk in Team Sport: A Preliminary Report
Source: Rev Bras Ortop (Sao Paulo). 2024 Apr 22;59(4):e584–9. doi: 10.1055/s-0043-1771531 (PMC11374406; doi:10.1055/s-0043-1771531)
Supplement: Related texts

# Research article: Gene COL5A1 e o risco de lesões do LCA no esporte de equipe: Um relatório preliminar

**Keywords:** atletas, ligamento cruzado anterior, polimorfismo de nucleotídeo único, ruptura, traumatismos em atletas

## Abstract

**Objetivo:** O objetivo deste estudo foi examinar a relação entre os polimorfismos do comprimento do fragmento de restrição (RFLP) BstUI C/T (rs 12722) e RFLP DpnII B1/B2 (rs 13946) COL5A1 e a ruptura do ligamento cruzado anterior (LCA) em atletas de esportes coletivos.

**Métodos** Sessenta e oito atletas de esportes coletivos (n = 36 mulheres e n = 32 homens) com ruptura do LCA (RLCA) sem contato ocorreram durante práticas esportivas (Grupo RLCA) e 42 jogadores saudáveis (n = 20 mulheres e n = 22 homens) (Grupo Controle) participaram do estudo. O DNA genômico foi extraído do swab bucal com o método salting out. Todas as amostras foram genotipadas para os polimorfismos rs12722 e rs13946 por reação em cadeia da polimerase (PCR) e análise de enzimas de restrição.

**Resultados** Nenhuma diferença significativa foi encontrada entre os grupos RLCA e Controle em idade, altura, peso corporal, índice de massa, prática esportiva (horas/semana) e distribuição de gênero entre os diferentes esportes coletivos. O grupo controle teve carreiras esportivas mais longas (p< 0,005). As distribuições de frequência dos polimorfismos de nucleotídeos COL5A1 DpnII estavam em equilíbrio de Hardy-Weinberg (EHW) em ambos os grupos (p do teste de Hardy-Weinberg (HW) > 0,005). As frequências genotípicas de COL5A1 BstUI RFLP C/C foram menores no grupo RLCA em comparação com o grupo Controle (p do teste HW = 0,001). Os genótipos combinados CC, B1B1 mostraram um efeito protetor contra a ruptura do LCA (OR = 83,3 / 16,7 = 5).

**Conclusões** O gene COL5A1 pode ser um dos fatores genéticos associados à RLCA em esportes coletivos.

## Introdução

A ruptura do ligamento cruzado anterior (RLCA) é uma lesão incapacitante frequente em atletas, que causa instabilidade articular do joelho. Nos EUA, a incidência estimada de RLCA varia entre 100.000 e 200.000/ano. 1 A cirurgia reconstrutiva permite a prática desportiva e uma melhor qualidade de vida. Na Itália, a incidência de cirurgia de reconstrução do LCA é de 21-33 por ano a cada 100.000 pessoas, com incidência entre 0,16 e 2,04 procedimentos por 100.000 indivíduos menores de 15 anos. 2 3

Apesar da reconstrução cirúrgica do ligamento, cerca de 79% desses pacientes desenvolvem osteoartrite do joelho e 20% sofrem uma nova lesão nos próximos 2 anos. 4

As RLCA esportivas podem ser lesões de contato (com um companheiro de equipe ou adversário), mais frequentes em esportes de contato como futebol ou basquete, 5 ou lesões sem contato. Em um período de observação de 10 anos, Majewski et al. 6 observaram que 60% das lesões esportivas do joelho tratadas em seu hospital eram lesões do LCA relacionadas ao voleibol. Agel et al. 7 constataram que 14% das lesões no voleibol ocorrem por não contato.

Ao longo de um período de 16 anos de registro de lesões colegiais, Hootman et al. 8 relataram uma taxa de lesões por 1.000 exposições de atletas de 0,07 e 0,09 para jogadores masculinos de basquete e futebol. Eles relatam 0,23, 0,28 e 0,09 para jogadores de basquete, futebol e vôlei, respectivamente.

Os fatores de risco para lesão do LCA são classificados como exógenas (por exemplo, a superfície) e endógenas, como fatores genéticos. 9 Em distúrbios hereditários do colágeno, a genética influencia a frouxidão dos tecidos 10 : frouxidão ligamentar generalizada e hiperextensão demonstraram aumentar significativamente o risco de lesão do LCA em atletas do sexo feminino. 11 Mutações no gene COL5A1 causam a forma clássica da síndrome de Ehlers-Danlos (SED), caracterizada por hipermobilidade articular envolvida em entorses, luxação/subluxação e osteoartrite precoce. 12 Conforme descrito por Mokone et al. 13 , o gene COL5A1 contém RFLP de BstUI (rs 12722) e DpnII (rs13946) dentro de sua região não traduzida (UTR) 3'. O polimorfismo funcional do sítio de ligação da proteína 1 (Sp1) de especificidade COL5A1 - BstUI RFLP C / T está positivamente correlacionado com lesões tendíneas e ligamentares, especialmente em indivíduos caucasianos. 14 15 Assim, um estudo com jogadores de futebol descobriu que o genótipo T/T do COL5A1 BstUI RFLP mostrou uma tendência a uma maior gravidade das lesões musculoesqueléticas em relação aos portadores individuais do genótipo CC. 15

O objetivo deste estudo foi correlacionar os polimorfismos BstUI RFLP e DpnII RFLP do gene COL5A1 com ACLR em atletas de voleibol, basquete e futebol.

## Materiais e métodos

## Participantes

Participaram do estudo 68 jogadores caucasianos (n = 36 mulheres e n = 32 homens) com RLCA ocorrido durante a prática esportiva (Grupo RLCA) e 42 jogadores saudáveis (n = 20 mulheres e n = 22 homens) (Grupo Controle). Todos praticavam esportes coletivos (vôlei, basquete e futebol) em times italianos. Os indivíduos deram consentimento informado para participar. Para a obtenção de informação demográfica e dados relativos à prática desportiva, cada atleta completou um questionário autoaplicável. O Grupo RLCA (32 atletas praticantes de Voleibol, 19 Basquetebol e 17 Futebol) forneceu informações sobre os mecanismos e data da lesão do LCA.

O estudo foi aprovado pelo Conselho de Ética em Pesquisa da Universidade de Cagliari (Prot. PG/2017/1700) e foi realizado de acordo com os princípios éticos e humanos da pesquisa.

## Análise de DNA

O DNA genômico foi extraído de swab bucal, realizado na mucosa oral da bochecha, pelo método salting out, e amplificado por PCR padrão seguindo o protocolo sugerido por Galasso et al. 16

Os produtos de PCR foram submetidos a duas digestões enzimáticas: a digestão com BstUI produziu dois fragmentos para o alelo T (351 e 316 pb) e três fragmentos para o alelo C (316, 271 e 80 pb); a digestão com DpnII produziu um único fragmento para B2 (612 pb) e dois fragmentos (412 e 194 pb) para B1. Os fragmentos obtidos foram separados em gel de poliacrilamida 8% para BstUI e gel de agarose 2% para DpnII e visualizados com coloração Syber Safe.

## Análise estatística

Todos os dados foram inicialmente inseridos em um banco de dados Excel (Microsoft, Redmond, Washington – Estados Unidos) e a análise foi realizada por meio do Statistical Package for the Social Sciences Windows, versão 15.0 (SPSS, Chicago, Illinois, EUA). A estatística descritiva consistiu na média ± desvio padrão (DP) para parâmetros com distribuição gaussiana (após confirmação com histogramas e teste de Kolgomorov-Smirnov).

A comparação entre os grupos foi realizada com ANOVA one-way para variáveis paramétricas contínuas ou teste Qui-quadrado ou teste exato de Fisher (se células <5) para variáveis de frequências.

Os testes EHW foram realizados considerando as frequências populacionais (p2 + 2pq + q2 = 1) e posteriormente relatados no SPSS, teste não paramétrico Qui-quadrado. Da mesma forma, o teste G-quadrado (teste G2) foi realizado. Um valor de p < 0,05 foi considerado estatisticamente significativo.

## Resultados

Nenhuma diferença significativa foi encontrada entre os grupos RLCA e Controle em idade, altura, peso, índice de massa corporal, prática esportiva (horas/semana) e distribuição de gênero entre os diferentes esportes coletivos). Os atletas do Grupo de controle têm carreiras esportivas mais longas (p < 0,005) ( Tabela 1 ).

**Tabela 1:** Características do Grupo com ruptura do LCA (RLCA) e Grupo Controle

	RLCA	Controle	*p*
Número	**68**	**42**	
Idade (anos)	**27.3 ± 6.4**	**28.5 ± 8.5**	0.406*
Altura (cm)	**173.0 ± 9.5**	**174.0 ± 11.4**	0.588*
Peso (kg)	**70.0 ± 11.8**	**70.2 ± 12.3**	0.945*
Índice de massa corporal (kg/cm ^2^ )	**23.30 ± 3.30**	**23.16 ± 2.99**	0.782*
Sexo (n° masculino/n° feminino)	**32 / 36**	**22 / 20**	**0.200^**
Esportes (vôlei; basquete; futebol)	**32; 19; 17**	**26; 8; 8**	**0.312^**
ESPORTE (anos)	**10.8 ± 4.9**	**16.8 ± 8.5**	0.001*
ESPORTE (horas/semana)	**7.7 ± 3.2**	**7.8 ± 4.0**	0.799*

Os valores são expressos como média ± desvio padrão ou um número (n) quando aplicável.

(*) Teste qui-quadrado Anova unidirecional (^).

As frequências genotípicas e alélicas de COL5A1 BstUI RFLPC/T e COL5A1 DpnII RFLP B1/B2 são mostradas na Tabela 2 . As distribuições de frequência genotípica dos polimorfismos de nucleotídeos COL5A1 DpnII e COL5A1 BstUI RFLP atendem ao EHW em ambos os grupos (p. valor> 0,05).

**Tabela 2:** Frequências Genotípicas do Grupo RLCA e do Grupo Controle - Verificação do Equilíbrio de Hardy-Weinberg

BstU	RLCA	Controle
**CC**	**58.90**	**22.22**
**CT**	**34.25**	**44.44**
**TT**	**6.85**	**33.33**
**MAF (T)**	**0.2397**	**0.5556**
***p***	**0.3881**	0.3274*
**DpnII**	**RLCA**	**Controle**
**B1B1**	**71.23**	**75.56**
**B1B2**	**26.03**	**20.00**
**B2B2**	**2.74**	**4.44**
**MAF (B2)**	**0.15**	**0.1444**
***p***	**0.556**	0.2060*

(*) Teste de Equilíbrio de Hardy-Weinberg.

O desequilíbrio de ligação foi testado com LDlink (Machiela e Chanock, 2015) 17 usando dados de 1000 genomas (1000 Genomes Project Consortium, 2015). Os dois polimorfismos de nucleotídeo único (SNPs) resultaram em um desequilíbrio de ligação para as populações europeias.

A distribuição do genótipo C/C e do alelo C do COL5A1 BstUI RFLP foi menor no Grupo RLCA em comparação com o Grupo Controle (teste pG2= 0.001- Tabela 3 ). Não foram encontradas diferenças significativas na distribuição do polimorfismo DpnII RFLP entre o Grupo RLCA e os controles ( Tabela 3 ). Finalmente, a combinação dos genótipos CC + B1B1 foi mais frequente nos controles do que no grupo RLCA, e associada a um efeito protetor (OR = 83,3 / 16,7 = 5). Os genótipos TT, B2B2 estavam ausentes entre os participantes.

**Tabela 3:** Frequências Genotípicas do Grupo RLCA e do Grupo Controle

RLCA		Controle	*p*
COL5A1 BstUI% (CC; CT; TT)	23,8;61,0; 79,2 (0.954)	76,2; 39,0; 20,8 (0.368)	0.001°
COL5A1 DpnII% (B1B1; B1B2; B2B2)	60,8;66,7; 50,0 (0.990)	39,2;33, 3; 50,0 (0.497)	0.952°

(°) Teste qui-quadrado.

## Discussão

Embora as alterações biológicas subjacentes ao aumento do risco de lesão esportiva do LCA ainda não tenham sido descobertas, a predisposição genética de atletas para rupturas do LCA é um conhecimento consolidado. 18 19

O gene COL5A1, localizado em 9q34.2-q34.3, contém 66 exons distribuídos em 150 kb de gDNA e codifica a cadeia 1 do colágeno tipo V. 20

O gene COL5A1 codifica uma cadeia proteica no colágeno tipo V, que se encontra em ligamentos e tendões, e mutações dentro dele são apontadas como causa responsável pelo aumento do risco de ruptura do LCA. 19 21

Examinamos a associação entre os polimorfismos COL5A1 rs12722 C/T (BstUI RFLP) e COL5A1 rs13946 B1/B2 (DpnII) individualmente e como haplótipos com risco de ruptura do ligamento cruzado anterior em atletas masculinos e femininos competindo em esportes coletivos de contato/sem contato.

Nossos resultados mostram uma frequência menor do genótipo COL5A1 BstUI C/C no Grupo RLCA em comparação com o grupo Controle. Nenhuma diferença significativa na distribuição de genótipos ou frequências alélicas de COL5A1 DpnII foi observada.

Posthumus et al. 21 encontraram um genótipo C/C sub-representado de COL5A1 BstUI RFLP em mulheres caucasianas, mas não em homens com rupturas de LCA diagnosticadas cirurgicamente recrutados de clubes esportivos e recreativos.

Em esquiadores amadores do sexo masculino, Stępień-Słodkowska et al. 22 não observaram diferenças significativas na distribuição genotípica ou nas frequências alélicas dos polimorfismos C/T C/T COL5A1 BstUI RFLP (rs 12722) e COL5A1 DpnII RFLP (rs 13946) entre o grupo RLCA e o grupo controle. Esses autores encontraram uma tendência de sub-representação do haplótipo C-T no grupo RLCA em comparação aos controles.

Luliska-Kuklik et al. 23 encontraram uma diminuição significativa na frequência no modelo dominante do genótipo C/C para o gene COL5A1 rs13946 em jogadores profissionais de futebol do sexo masculino com ruptura primária do LCA diagnosticada cirurgicamente.

Em nosso estudo, os atletas dos grupos RLCA e controle foram pareados por idade, altura, peso corporal, IMC, esporte e duração de sua carreira esportiva. Quanto mais longa a carreira esportiva, maior o risco de exposição ao trauma.

Além de apresentar maior frequência do genótipo C/C, os atletas do Grupo Controle têm uma carreira esportiva mais longa do que os do Grupo RLCA: com cautela devido ao pequeno número de sujeitos, hipotetizamos um efeito protetor do C Genótipo /C, especialmente se associado a B1B1 (CC, B1B1 OR protetor = 83,3 / 16,7 = 5).

Da mesma maneira, Posthumus et al. 24 encontraram um aumento significativo dependente da idade na distribuição do genótipo COL5A1 BstUI RFLP C/C no Grupo de atividade física do sexo masculino, assintomático para lesões musculoesqueléticas de tecidos moles (sem histórico relatado de lesões tendíneas). Segundo esses autores, prolongar o tempo de exposição ao risco extrínseco de lesão seleciona indivíduos geneticamente de baixo risco de lesão, que serão encontrados em maior número entre os assintomáticos mais velhos do que nos assintomáticos mais jovens.

O polimorfismo COL5A1 BstUI RFLP C/C pode desempenhar um papel no aumento da duração da carreira esportiva dos atletas.

Não está claro qual expressão fenotípica dos polimorfismos do gene COL5A1 pode estar associada a um risco aumentado de ruptura do LCA.

Na SED, várias mutações do gene COL5A1 ocorrem simultaneamente com características clínicas específicas. 10 O tecido conjuntivo desses pacientes apresenta alterações estruturais e frouxidão ligamentar causando diferentes graus de hipermobilidade articular.

A hipermobilidade tem sido implicada na lesão do LCA, 25 e a propriocepção articular dos membros inferiores é reduzida naqueles com síndrome de hipermobilidade articular benigna 26 : nenhuma correlação foi descrita entre os polimorfismos COL5A1 BstUI RFLP e COL5A1 DpnII com aumento da frouxidão ligamentar. Investigações futuras devem incluir a busca de uma associação entre alterações na mobilidade articular ou tensão ligamentar e a presença desses polimorfismos.

Em um estudo retrospectivo de associação caso-controle genético, O'Connell et al. 27 encontraram o genótipo COL5A1 C/C significativamente super-representado em atletas de triátlon e ultramaratona sem histórico de cãibras musculares associadas ao exercício (CMAE) em comparação com atletas com histórico de CMAE. A eficiência dos músculos flexores do joelho, dos músculos abdutores do quadril e dos músculos estabilizadores do tronco previne lesões do LCA. 28

De acordo com Collins and Posthumus, 29 o genótipo COL5A1 rs12722 TT está associado a uma produção excedente de colágeno V com maior risco de algumas lesões musculoesqueléticas devido a alterações estruturais das fibrilas de colágeno nas fibrilas de colágeno e alterações nas propriedades dos tecidos moles.

Laguette et al. 30 demonstraram maior estabilidade do mRNA COL5A1 (codificando para α1(V)) no genótipo C/T e especularam que pequenas mudanças na estabilidade do mRNA COL5A1, mesmo que dentro da faixa fisiológica normal, poderiam resultar em variação interindividual na fibrilogênese; portanto, uma vulnerabilidade diferente a lesões musculoesqueléticas dependentes de COL5A1. Teorizamos que, no grupo de estudo (Grupo RLCA), a frequente exposição do LCA a estresses anormais durante a prática de esportes coletivos revela a suscetibilidade relativa do LCA dos atletas com genótipo C/T.

Conforme relatado por Smith et al., 19 mais três fatores genéticos podem estar associados ao aumento da fragilidade ligamentar: 1) genótipo TT do COL1A1 Sp1, polimorfismo do sítio de ligação. O gene COL1A1 codifica uma cadeia de proteína dentro do colágeno tipo I, um importante componente estrutural dos ligamentos; 2) o genótipo AA do polimorfismo COL12A1 AluI (somente em mulheres). Esse gene codifica as cadeias de proteína no colágeno tipo XII, que se acredita regular o diâmetro das fibrilas nos ligamentos; 3) a região cromossômica 11q22, onde estão localizados vários genes de metaloproteinases da matriz de mediadores fisiológicos da clivagem e remoção do colágeno.

A dependência genética múltipla da susceptibilidade à ruptura do LCA pode sugerir alterações de varredura em múltiplos genes.

A principal limitação do presente trabalho é representada pelo pequeno número de atletas incluídos no estudo, o que impossibilitou a análise de diferenças entre os gêneros. O desenvolvimento de ferramentas clínicas ou laboratoriais que identifiquem indivíduos com grande risco para essa lesão musculoesquelética comum, facilitaria uma maior conscientização das pessoas sobre sua própria exposição a uma ruptura do LCA. Além disso, seria útil para definir a melhor escolha terapêutica após o diagnóstico de ruptura primária do LCA.

## Conclusões

O gene COL5A1 pode ser um dos fatores genéticos associados à RLCA em esportes coletivos. Os polimorfismos COL5A1 C/C fornecem um efeito protetor contra RLCA em ambos os sexos. Carreira esportiva mais longa ligada a uma frequência aumentada de COL5A1 BstUI RFLP C/C.
